# Author Correction: Myeloid lineage enhancers drive oncogene synergy in CEBPA/CSF3R mutant acute myeloid leukemia

**DOI:** 10.1038/s41467-022-30987-0

**Published:** 2022-06-16

**Authors:** Theodore P. Braun, Mariam Okhovat, Cody Coblentz, Sarah A. Carratt, Amy Foley, Zachary Schonrock, Brittany M. Curtiss, Kimberly Nevonen, Brett Davis, Brianna Garcia, Dorian LaTocha, Benjamin R. Weeder, Michal R. Grzadkowski, Joey C. Estabrook, Hannah G. Manning, Kevin Watanabe-Smith, Sophia Jeng, Jenny L. Smith, Amanda R. Leonti, Rhonda E. Ries, Shannon McWeeney, Cristina Di Genua, Roy Drissen, Claus Nerlov, Soheil Meshinchi, Lucia Carbone, Brian J. Druker, Julia E. Maxson

**Affiliations:** 1 Knight Cancer Institute, Oregon Health & Science University, Portland, OR 97239 USA; 2 Division of Hematology and Medical Oncology, Oregon Health & Science University, Portland, OR 97239 USA; 3 Knight Cardiovascular Institute, Oregon Health & Science University, Portland, OR 97239 USA; 4 Program in Molecular and Cellular Biology, Oregon Health & Science University, Portland, OR 97239 USA; 5 Computational Biology Program, Oregon Health & Science University, Portland, OR 97239 USA; 6 Division of Bioinformatics and Computational Biology, Oregon Health & Science University, Portland, OR 97239 USA; 7 Clinical Research Division, Fred Hutchinson Cancer Research Center, 1100 Fairview Ave N, Seattle, WA 98109 USA; 8 MRC Molecular Haematology Unit, MRC Weatherall Institute of Molecular Medicine, John Radcliffe Hospital, Headley Way, Oxford, OX3 9DS UK; 9 Division of Pediatric Hematology/Oncology, University of Washington, Seattle, WA 98195 USA; 10 Department of Molecular and Medical Genetics, Oregon Health & Science University, Portland, OR 97239 USA; 11 Howard Hughes Medical Institute, Portland, OR USA

**Keywords:** Leukaemia, Cancer genomics

Correction to: *Nature Communications* 10.1038/s41467-019-13364-2, published online 29 November 2019.

Since the publication of this work, Brittany M. Curtiss has changed their name from Brittany M. Smith. This has now been amended.

