# Supplementary material for: Myeloid lineage enhancers drive oncogene synergy in CEBPA/CSF3R mutant acute myeloid leukemia
Source: Nat Commun. 2019 Nov 29;10:5455. doi: 10.1038/s41467-019-13364-2 (PMC6884457; doi:10.1038/s41467-019-13364-2)
Supplement: Supplementary file 2 — Supplementary Information [file 41467_2019_13364_MOESM2_ESM.pdf]

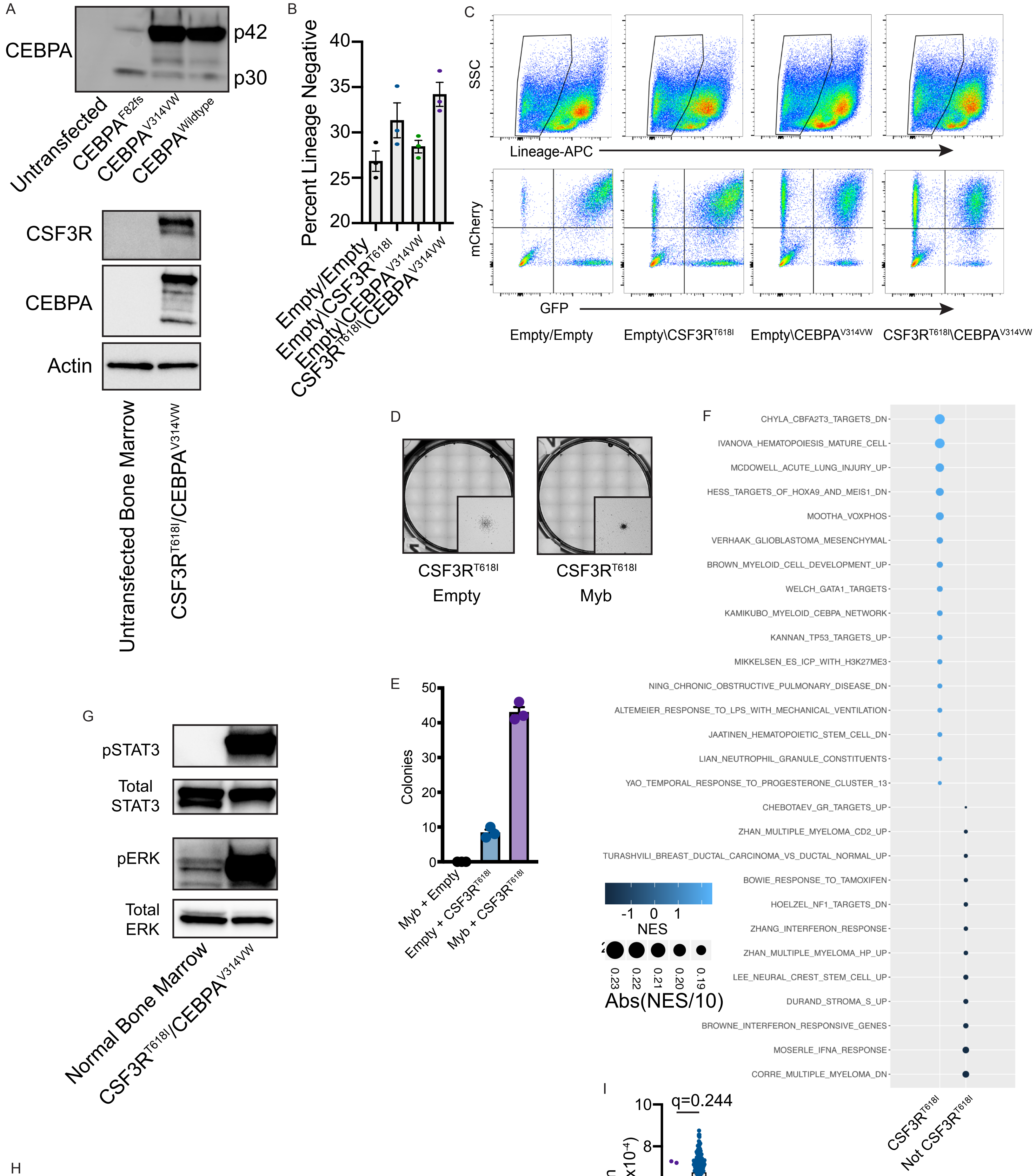

H

DE Gene Group	Total	number of genes with CEBPA peak within 10Kb	% genes with CEBPA peak within 10Kb	mean distance to CEBPA peaks	empirical p-value (observed mean distance compared to mean distance for randomly selected genes)
CEPBA-Down	220	157	71.4	816,455.60	<0.001
CEPBA-Up	425	239	56.2	3,106,105	0.113
CSF3R_Down	204	116	56.9	4,567,706	0.712
CSF3R_Up	372	225	60.5	2,132,837	0.009
Interacting	569	408	71.7	1,602,495	<0.001

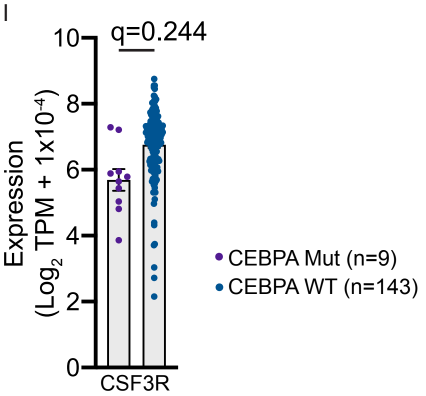

Supplementary Figure 1.

Supplementary Figure 1. CEBPA Mutations Block Myeloid Differentiation.
A. Western blot for CEBPA in K562 cells expressing CEBPA^{F82fs} or CEBPA^{V314VW} and of CEBPA and CSF3R in normal mouse bone marrow compared with a cell line derived from CSF3R^{T618I} and CEBPA^{V314VW} colony assay. **B.** Flow cytometric quantification of lineage negative cells sorted for RNA seq in Figure 2. **C.** Example gaiting for B. **D.** Colony assay from mouse bone marrow transduced with CSF3R^{T618I} and either Empty Vector or Myb (representative images). **E.** Quantification of colony assays from bone marrow transduced with CSF3R^{T618I} and either Empty Vector or Myb (n=3/group). **F.** Full GSEA results from Figure 2. All gene sets with q <0.05 shown. **G.** Western blot demonstrating activation of STAT3 and MAPK/ERK in CSF3R/CEBPA murine AML blasts. **H**. Permutation analysis of CEBPA ChIP-seq data and DE gene subsets showing enrichment of CEBPA peaks. p values determined via Fisher's exact test. **I.** Expression of CSF3R in CEBPA mutant and wild type pediatric AML. q values calculated with DEseq2 and corrected for multiple comparisons using the Benjamini-Hochberg method. Values in bar graphs are represented as mean with error bars representing SEM. Source data are provided as a Source Data file.

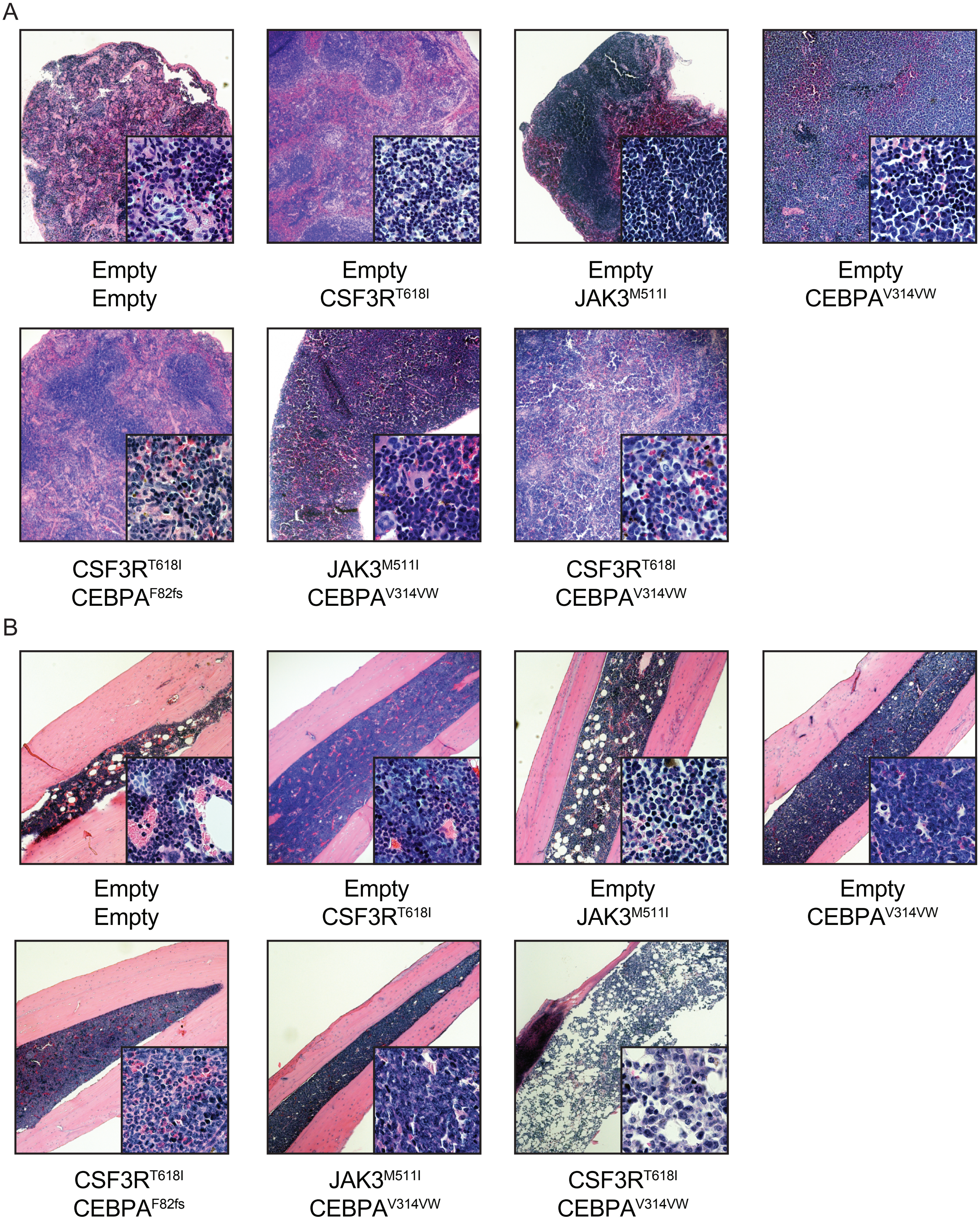
A
Empty
Empty
Empty
CSF3R^{T618I}
Empty
JAK3^{M511I}
Empty
CEBPA^{V314VW}
CSF3R^{T618I}
CEBPA^{F82fs}
JAK3^{M511I}
CEBPA^{V314VW}
CSF3R^{T618I}
CEBPA^{V314VW}
B
Empty
Empty
Empty
CSF3R^{T618I}
Empty
JAK3^{M511I}
Empty
CEBPA^{V314VW}
CSF3R^{T618I}
CEBPA^{F82fs}
JAK3^{M511I}
CEBPA^{V314VW}
CSF3R^{T618I}
CEBPA^{V314VW}

Figure S2.

Supplementary Figure 2. Histologic Assessment of Leukemia Development
A. Example images from the spleens of animals transplanted with the oncogene combinations described in Figure 3 taken at the experimental endpoint (either moribund or at experiment end for Empty/Empty). **B.** Example images from the spleens of animals transplanted with the oncogene combinations described in Figure 3 taken at the experimental endpoint (either moribund or at experiment end for Empty/Empty). Low resolution images are taken at 5X while high resolution images are at 63X.

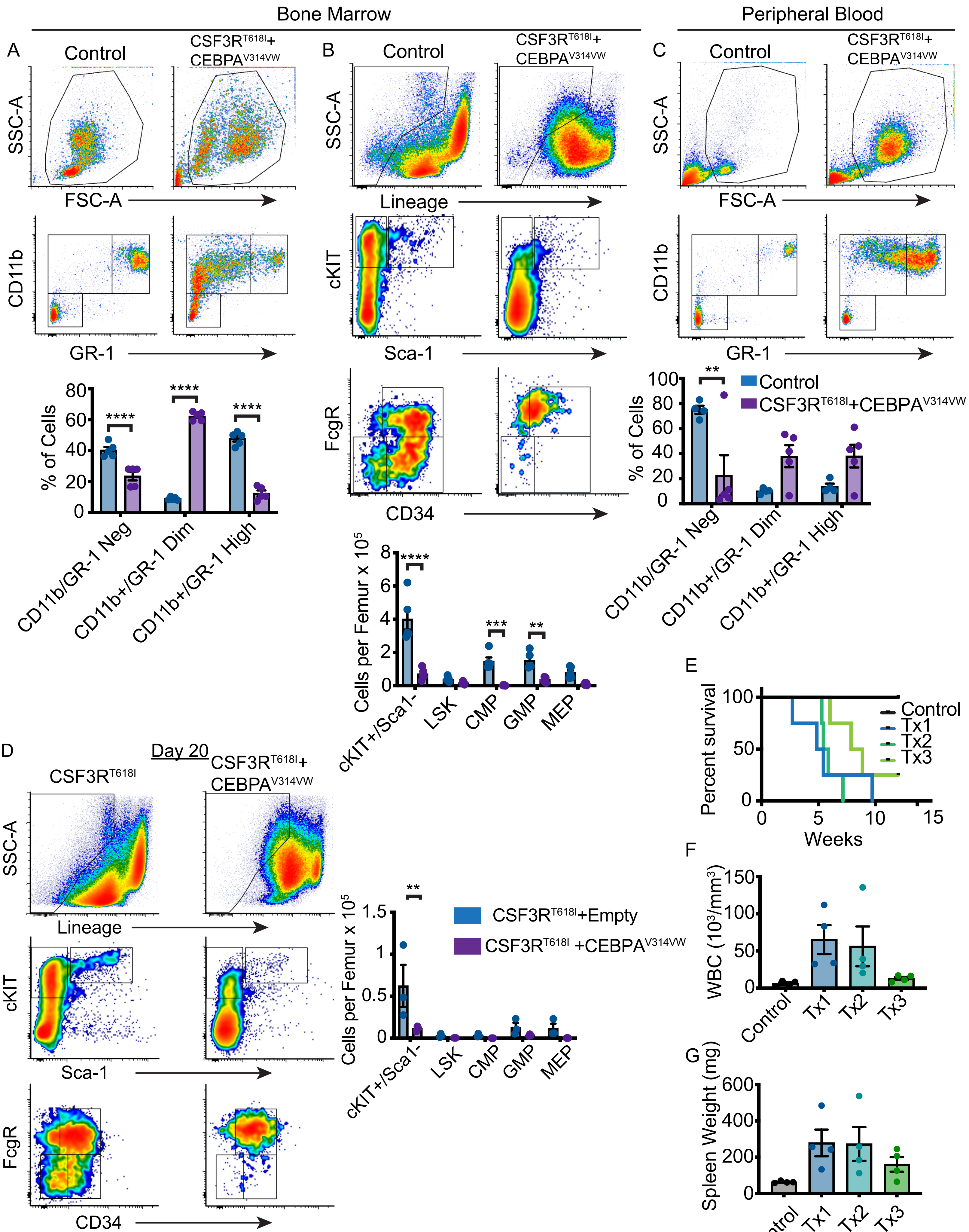

Supplementary Figure 3.

Supplementary Figure 3. CSF3R^{T618I} and CEBPA^{V314VW} combine to produce an immature leukemia with replacement of normal hematopoiesis, while CEBPA^{F82fs} does not modify disease phenotype.
A. Myeloid differentiation assessed by flow cytometry from bone marrow of BALB/c mice transplanted with 100,000 bone marrow cells transduced with CSF3R^{T618I} and CEBPA^{V314VW} at 14 days post-transplant as compared with control mice (Mice transplanted with empty vector are pancytopenic at day 14; n=5/group). **B**. Stem and progenitor cell populations from groups in A (n=5/group). **C.** Myeloid differentiation in the peripheral blood as assessed by flow cytometry for groups in A (n=4-5/group). **D.** Flow cytometric assessment of stem/progenitor cell populations at day 20 post-transplant in BALB/c mice transplanted with 10,000 cells transduced with CSF3R^{T618I} and CEBPA^{V314VW} compared with mice receiving 100,000 cells transduced with CSF3R^{T618I} and empty vector (At this time point, control mice had recovered from transplant-induced pancytopenia allowing direct comparison, same cohort as Figure 4B-F, n=3/group). **E.** Survival after serial transplantation of 100,000 bone marrow cells from moribund primary recipients. Successive serial transplantation from moribund recipients for 3 serial transplantations (n=4-5/group). **F.** Terminal WBC counts from each round of serial transplantation. **G.** Spleen weight from each round of serial transplantation. Values in bar graphs are represented as mean with error bars representing SEM. Statistical significance in all panels assessed by 2-way ANOVA with Sidak's post-test, **: $p<0.01$, ***: $p<0.001$, ****: $p<0.0001$. Source data are provided as a Source Data file.

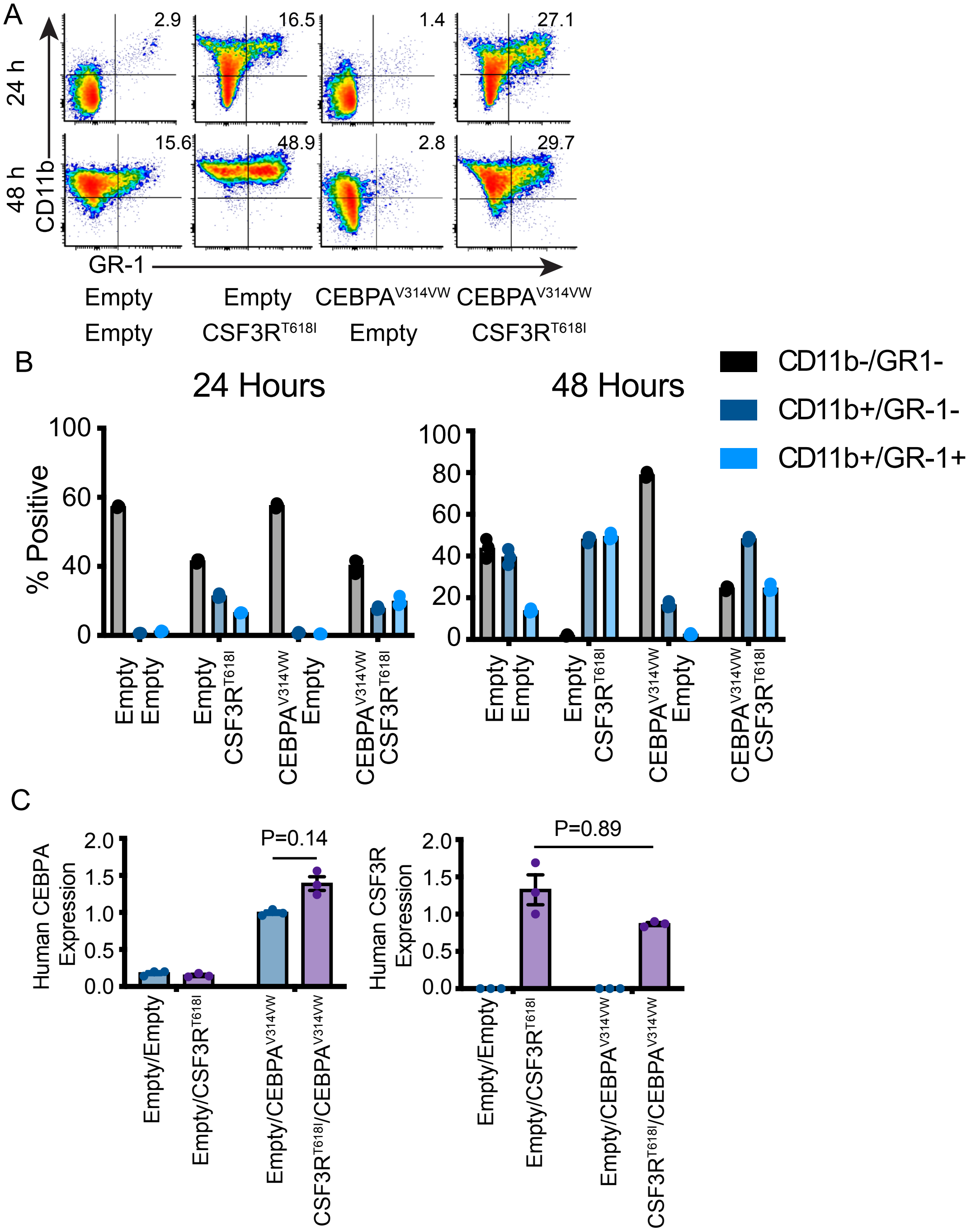

Supplementary Figure 4.

Supplementary Figure 4. CSF3R^{T618I} and CEBPA^{V314VW} have opposing effects on myeloid differentiation in HoxB8 cells.

A. Expression of CD11b and GR-1 by flow cytometry in murine HoxB8-ER cells transduced with empty vector, CSF3R^{T618I}, CEBPA^{V314VW} or the oncogene combination 24-48 hours after estrogen withdrawal. **B.** Quantification of percent of murine HoxB8-ER cells expressing GR-1 and CD11b as measured by flow cytometry after estrogen withdrawal. Cells were transduced with empty vector, CSF3R^{T618I}, CEBPA^{V314VW} or both oncogenes in combination. **C.** Human CEBPA and CSF3R expression in HoxB8-ER cells from 3A, 24 hours after estrogen withdrawal as measured by Q-RT PCR. Statistical significance assessed by two-way ANOVA with Sidak's post-test. (n=3/group). Values in bar graphs are represented as mean with error bars representing SEM. Source data are provided as a Source Data file.

A

Human AML Enhancers	Left p value (Depleted)	Right p value (Enriched)
CEBPA Wild Type	0	1
CEBPA Mutant	1	3.3219E-73

B

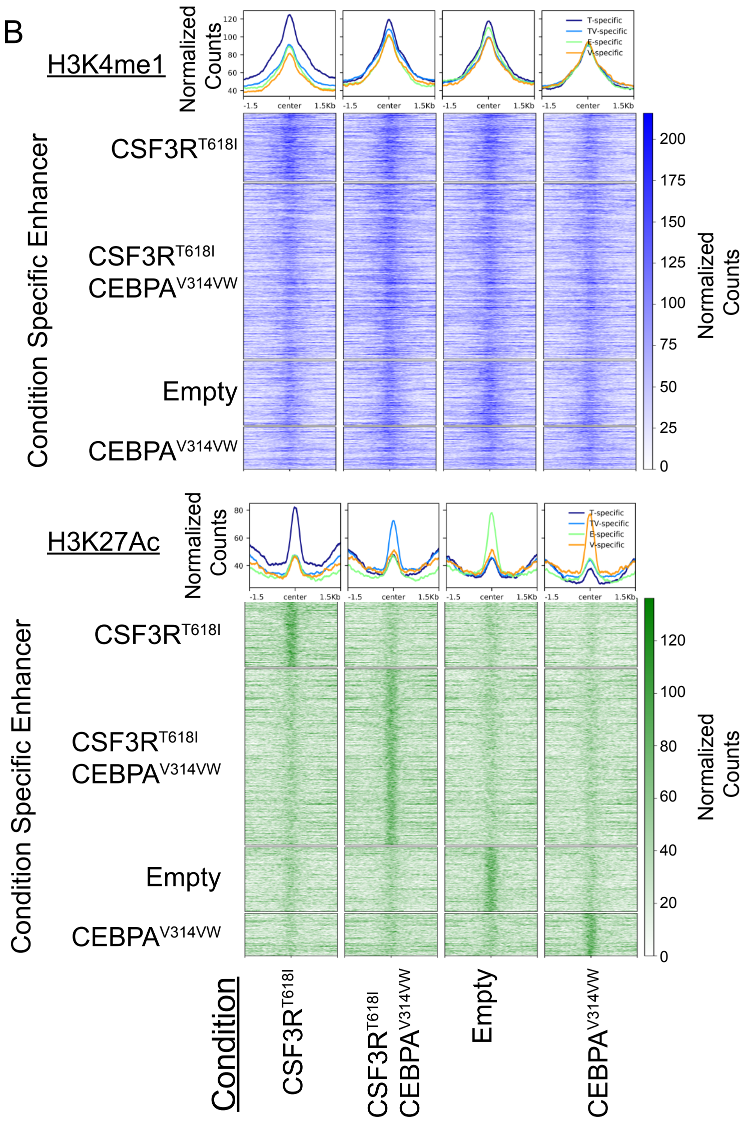

C

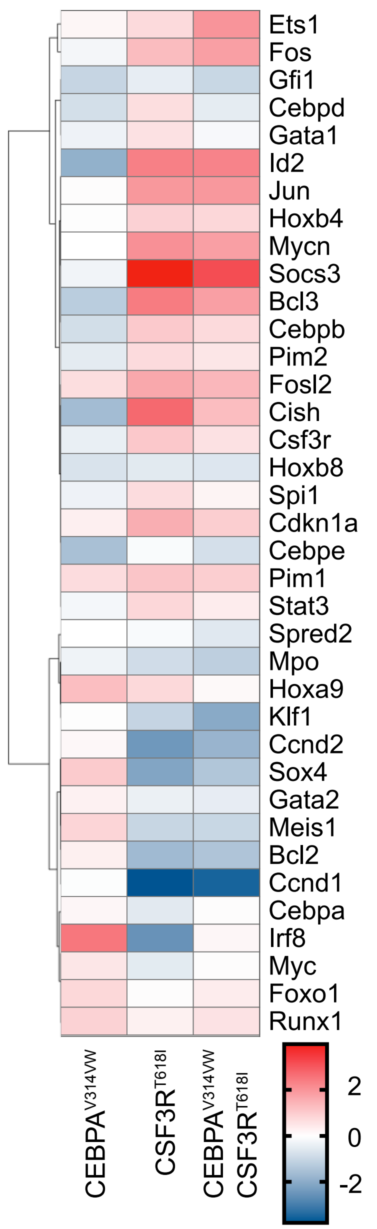

D

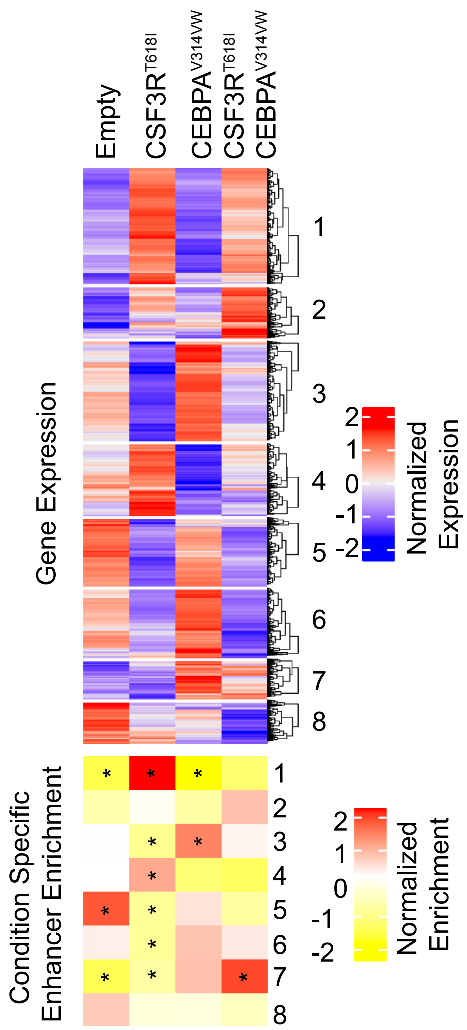

Supplementary Figure 5.

Supplementary Figure 5. CEBPA Mutation Blocks Activation of $CSF3R^{T618I}$ Specific Enhancers. **A.** Overlap of enhancers identified in human CEBPA mutant AML or CEBPA WT AML with mouse $CSF3R^{T618I}$/$CEBPA^{V314VW}$ specific enhancers measured by Bedtools using Fishers-exact test. Right sided P value indicative of enrichment, left sided p value indicative of depletion. **B.** Heatmaps of H3K4me1 and H3K27Ac across each group of condition specific enhancers in all 4 treatment conditions**. C.** Confirmation of expression profile for specific genes identified in microarray analysis, performed by Taqman low density Q-PCR array, normalized to *GusB* and displayed as a relative quantity compared with empty vector control. All genes with differential expression ($q<0.05$) in at least one pairwise comparison shown. **D.** Unsupervised hierarchical clustering of differentially expressed genes (as assessed by microarray) and associated enrichment analysis for condition specific enhancers. Bottom heat map displays normalized Pearson residual values from χ^2 test for each category of condition specific enhancer and gene expression cluster. P values adjusted by the method of Holm-Bonferroni. Cluster numbers appear on the right side and *: $p<0.05$. Source data are provided as a Source Data file.

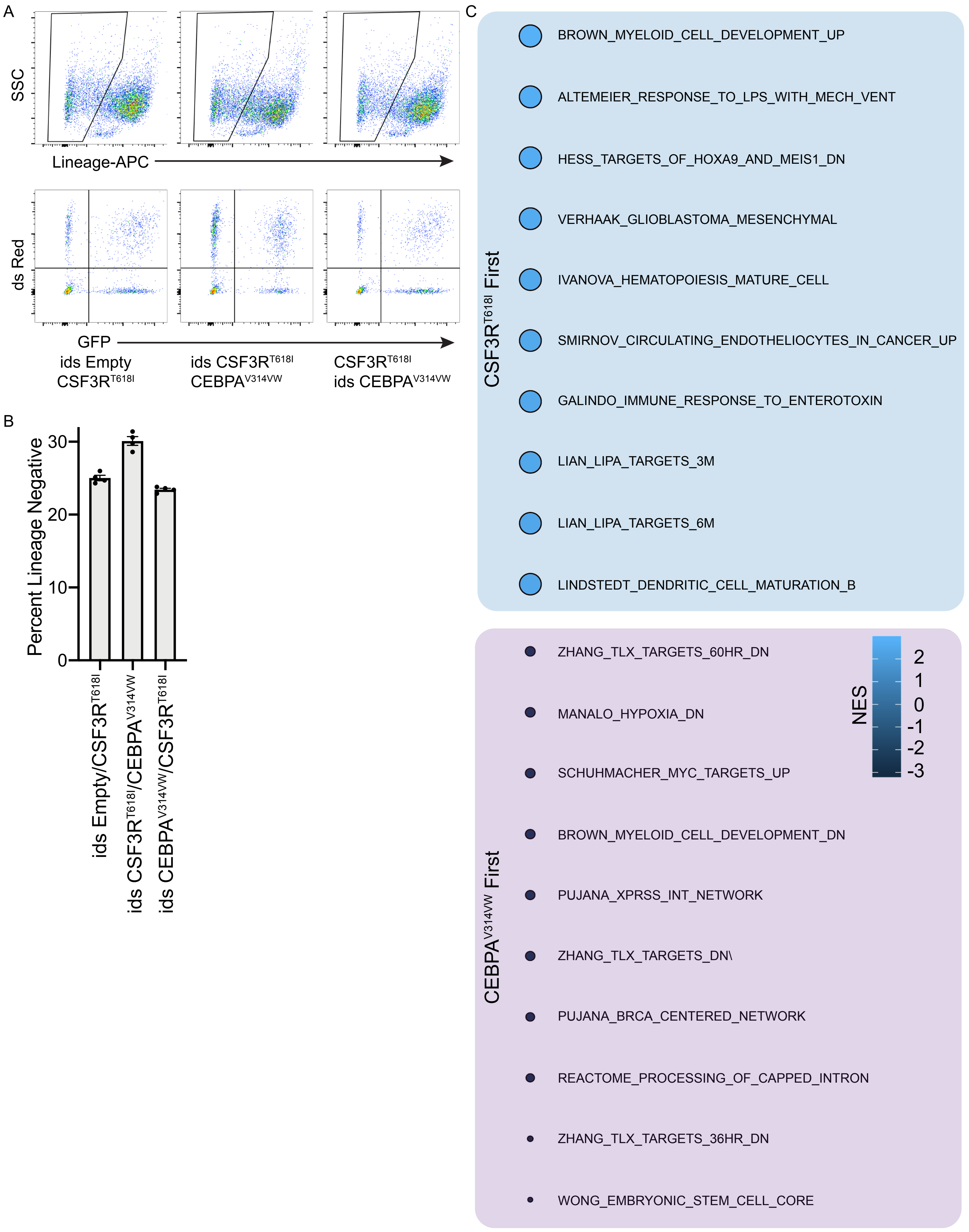

Supplementary Figure 6.

Supplementary Figure 6. CEBPA First Blocks Myeloid Differentiation Gene Signatures Associated with CSF3R^{T618I}.
A. Flow sorting strategy for RNA seq in Figure 6. **B.** Quantification of lineage negative cell population from A. **C.** Gene Set Enrichment Analysis results from RNA-seq data Figure 6. C2 collection gene sets with q <0.05 enriched in CSF3R First and enriched in CEBPA First. GSEA p-value calculated by empirical permutation test and FDR adjusted. Source data are provided as a Source Data file.

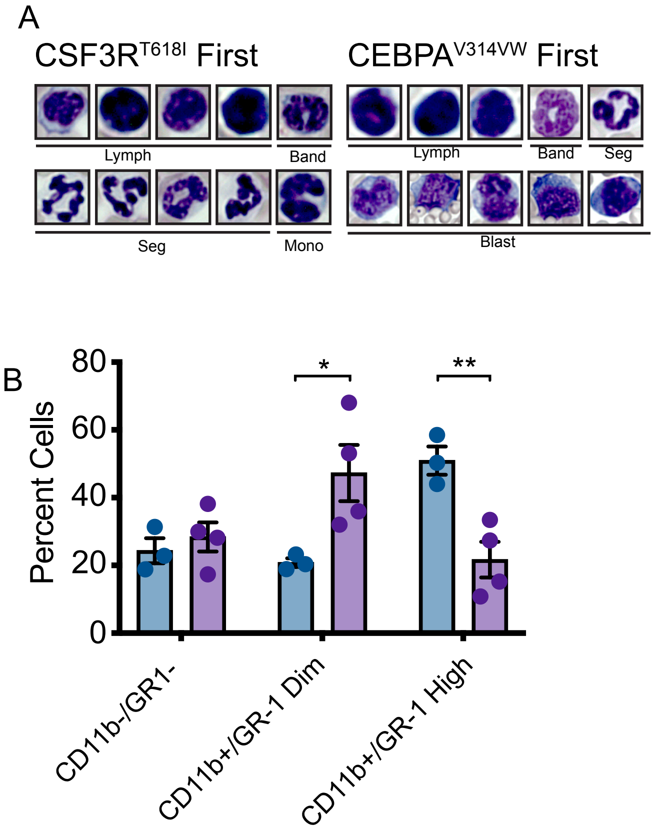

Supplementary Figure 7.

Supplementary Figure 7. CEBPA^{V314VW} must precede CSF3R^{T618I} to produce a myeloid malignancy with differentiation block and cell cycle dysregulation.
A. Representative images of cells for manual differential (representative of 3 animals/group). **B.** Quantification of flow cytometry from Figure 7 (n=3/group). Values in bar graphs are represented as mean with error bars representing SEM. *<0.05, **: p<0.01 by ANOVA with Sidak's post-test. Source data are provided as a Source Data file.

SUPPLEMENTARY TABLES

Supplementary Table 1: PCR Primers for Site Directed Mutagenesis.

Gene	5' Primer	3' Primer
CEBPA^{F82fs}	ctggccgacctgttgatcccagcacagcc g	cggctgtgctgggatcaacaggtcggcc ag
CEBPA^{V314VW}	gtcagctccagccacaccttctgctgcgtct	agacgcagcagaaggtgtggctggagc tgac
CEBPA^{K313KR}	cagctccagcacccttttctgctgcgtctcc	ggagacgcagcagaaaagggtgctgg agctg
JAK3^{M511I}	ggatcttgtgaaatgttatctgactcagctgg tattgg	ccaataccagctgagtcagataacatttc acaagatcc

Supplementary Table 2: Taqman Primer Probes.

Gene	Taqman Probe ID
Bcl2	Mm00477631_m1
Bcl3	Mm00504306_m1
Bcl6	Mm00477633_m1
Fos	Mm00487425_m1
Ccnd2	Mm00438070_m1
Cdkn1a	Mm04205640_g1
Human CEBPA	Hs00269972_s1
Mouse Cebpa	Mm00514283_s1
Cebpb	Mm00843434_s1
Cebpd	Mm00786711_s1
Cebpe	Mm02030363_s1
Cish	Mm01230623_g1
18s rRNA	Hs99999901_s1
Myc	Mm00487804_m1
Csf3r	Mm00432735_m1
Ccnd1	Mm00432359_m1
Elk1	Mm00468233_g1
Ets1	Mm01175819_m1
Etv6	Mm01261325_m1
Fosl2	Mm00484442_m1
Foxo1	Mm00490671_m1
Foxo3	Mm01185722_m1
Gata1	Mm01352636_m1
Gata2	Mm00492301_m1
Gfi1	Mm00515853_m1
Hck	Mm01241463_m1
Hoxa10	Mm00439368_m1
Hoxa9	Mm00439364_m1
Hoxb4	Mm00657964_m1
Hoxb8	Mm00516096_m1
Mecom	Mm00491303_m1

Meis1	Mm00487664_m1
Mpo	Mm01298424_m1
Mycn	Mm00476449_m1
Pbx1	Mm04207617_m1
Pla2g7	Mm00479105_m1
Pim1	Mm00435712_m1
Pim2	Mm00454579_m1
Spi1	Mm00488140_m1
Stfa1	Mm01973758_m1
Tnfsf11	Mm00441906_m1
Runx1	Mm01213404_m1
Socs3	Mm00545913_s1
Sox4	Mm00486320_s1
Spred2	Mm01223872_g1
Stat3	Mm01219775_m1
Stat5a	Mm03053818_s1
Gusb	Mm01197698_m1
Gapdh	Mm99999915_g1

Supplementary Table 3: Antibodies.

Antibody	Manufacturer	Cat #	Dilution
PE Rat Anti-Mouse GR-1 (RB6-8C5)	BD	553128	1:500
PE-Cy7 Rat Anti-Mouse CD11b (M1/70)	BD	561098	1:500
PE-Cy7 Rat Anti-Mouse CD117 (2B8)	BD	558163	1:200
PE Rat Anti Mouse Sca-1 (D7)	BD	553108	1:100
APC Mouse Lineage Cocktail	BD	558074	1:20
BV421 Rat Anti-Mouse CD34 (RAM34)	BD	562608	1:20
PerCP-e710 Rat Anti-Mouse CD16/32 (93)	eBiosciences	46-0161-80	1:50
e450 Rat Anti-Mouse CD34 (RAM34)	eBiosciences	48-0341-80	1:20
Polyclonal Rabbit Anti-H3K4me1	Abcam	ab8895	2 μg

Polyclonal Rabbit Anti-H3K4me3	Abcam	ab8580	1 μg
Polyclonal Rabbit Anti-H3K27ac	Abcam	ab4729	1.5 μg
Rabbit anti Phosphorylated Stat3	Cell Signaling Technologies	9131	1:1000
Rabbit anti Total Stat3	Cell Signaling Technologies	9139	1:1000
Rabbit anti p-p42/44 MAPK T202/Y204	Cell Signaling Technologies	4370	1:1000
Rabbit anti Total p42/44 MAPK	Cell Signaling Technologies	4695	
Mouse anti CSF3R	R&D Systems	38043	1:1000
Rabbit anti actin	Cell Signaling Technologies	8457	1:5000
Rabbit anti CEBPA	Cell Signaling Technologies	D856F10	1:1000
