# Supplementary material for: Myeloid lineage enhancers drive oncogene synergy in CEBPA/CSF3R mutant acute myeloid leukemia
Source: Nat Commun. 2019 Nov 29;10:5455. doi: 10.1038/s41467-019-13364-2 (PMC6884457; doi:10.1038/s41467-019-13364-2)
Supplement: Supplementary file 3 — Description of Additional Supplementary Files [file 41467_2019_13364_MOESM3_ESM.pdf]

Description of Additional Supplementary Files

File Name: Supplementary Data File 1
Description: RNA Sequencing Results for Simultaneous Oncogene Introduction.

File Name: Supplementary Data File 2
Description: RNA Sequencing Results for TARGET Pediatric AML Samples.

File Name: Supplementary Data File 3
Description: Activated Enhancer Catalog from Differentiating HoxB8 Cells.

File Name: Supplementary Data File 4
Description: Gene Ontology Analysis for Condition Specific Enhancers.

File Name: Supplementary Data File 5
Description: Microarray Results from Differentiating HoxB8-ER Cells.

File Name: Supplementary Data File 6
Description: RNA Sequencing Analysis for Ordered Oncogene Introduction.
