# Supplementary material for: Myeloid lineage enhancers drive oncogene synergy in CEBPA/CSF3R mutant acute myeloid leukemia
Source: Nat Commun. 2019 Nov 29;10:5455. doi: 10.1038/s41467-019-13364-2 (PMC6884457; doi:10.1038/s41467-019-13364-2)
Supplement: Supplementary file 10 — Reporting Summary [file 41467_2019_13364_MOESM10_ESM.pdf]

nature research

Corresponding author(s): Julia E. Maxson

Last updated by author(s): May 23, 2019

Reporting Summary

Nature Research wishes to improve the reproducibility of the work that we publish. This form provides structure for consistency and transparency in reporting. For further information on Nature Research policies, see Authors & Referees and the Editorial Policy Checklist.

Statistics

For all statistical analyses, confirm that the following items are present in the figure legend, table legend, main text, or Methods section.

n/a	Confirmed	
☐	☒	The exact sample size (*n*) for each experimental group/condition, given as a discrete number and unit of measurement
☐	☒	A statement on whether measurements were taken from distinct samples or whether the same sample was measured repeatedly
☐	☒	The statistical test(s) used AND whether they are one- or two-sided<br>*Only common tests should be described solely by name; describe more complex techniques in the Methods section.*
☒	☐	A description of all covariates tested
☐	☒	A description of any assumptions or corrections, such as tests of normality and adjustment for multiple comparisons
☐	☒	A full description of the statistical parameters including central tendency (e.g. means) or other basic estimates (e.g. regression coefficient) AND variation (e.g. standard deviation) or associated estimates of uncertainty (e.g. confidence intervals)
☐	☒	For null hypothesis testing, the test statistic (e.g. *F*, *t*, *r*) with confidence intervals, effect sizes, degrees of freedom and *P* value noted<br>*Give P values as exact values whenever suitable.*
☒	☐	For Bayesian analysis, information on the choice of priors and Markov chain Monte Carlo settings
☒	☐	For hierarchical and complex designs, identification of the appropriate level for tests and full reporting of outcomes
☒	☐	Estimates of effect sizes (e.g. Cohen's *d*, Pearson's *r*), indicating how they were calculated

Our web collection on statistics for biologists contains articles on many of the points above.

Software and code

Policy information about availability of computer code

Data collection	BD FACSDiva v8.0.1, STEMvision Acquisition Application v1.0, Affymetrix Command Console v3.1.1, HiSeq Control Software v1.5.15.1 AI QuantsudioSoftware v6.0, IlluminaNextSeq System Suite v2.2.0
Data analysis	Affymetrix Transcript Analysis Console v4.0, HOMER v4.10, FCSExpress v6.0, Trimmomatic v0.32, STAR v2.6.1, DEseq2 v3.8, Gene Set Enrichment Analysis v2.0, Samtools v1.9, FastQC v0.11.8, ChromHMM v1.17, BEDTools v2.27.0, BEDOPS v2.4.7, Prism v7.0

For manuscripts utilizing custom algorithms or software that are central to the research but not yet described in published literature, software must be made available to editors/reviewers. We strongly encourage code deposition in a community repository (e.g. GitHub). See the Nature Research guidelines for submitting code & software for further information.

Data

Policy information about availability of data

All manuscripts must include a data availability statement. This statement should provide the following information, where applicable:

- Accession codes, unique identifiers, or web links for publicly available datasets
- A list of figures that have associated raw data
- A description of any restrictions on data availability

The accession numbers for all genomic data for RNA-seq, ChIP-seq and microarray data reported in Figure 1, Figure 3 and Figure 4 reported in this paper is GSE122166.

Field-specific reporting

Please select the one below that is the best fit for your research. If you are not sure, read the appropriate sections before making your selection.

☒ Life sciences ☐ Behavioural & social sciences ☐ Ecological, evolutionary & environmental sciences

For a reference copy of the document with all sections, see nature.com/documents/nr-reporting-summary-flat.pdf

Life sciences study design

All studies must disclose on these points even when the disclosure is negative.

Sample size	Sample size for in vitro experiments was selected based on prior experience with techniques utilized and familiarity with associated variance. In vivo studies were powered to detect a 50% difference in median survival.
Data exclusions	Data from high throughput drug screening was excluded if the following criteria were met: Drugs that were subsequently found to have more than a 10-fold change in IC50 in confirmatory studies were removed from the final screen results. Compounds that reached an IC50, but subsequently had 2 or more points >75% viability at higher concentrations, were excluded. Compounds that failed to drop viability below 25% were excluded.
Replication	All in vitro experiments reported as representative (ie without biological replicates) were reproduced in a minimum of 3 separate experiences.
Randomization	For in vitro experiments, all sample groups were evenly distributed between analysis groups (sequencing flow cells, qPCR plates). As body weight was utilized as an experimental endpoint for survival studies, starting body weights were normalized across treatment groups. Animals were not distributed into experimental groups based on other criteria.
Blinding	Colony assay experiments were counted by a blinded observer. Blinding was not possible in animal experiments due to the same laboratory staff being responsible for experimental setup, monitoring and drug administration.

Reporting for specific materials, systems and methods

We require information from authors about some types of materials, experimental systems and methods used in many studies. Here, indicate whether each material, system or method listed is relevant to your study. If you are not sure if a list item applies to your research, read the appropriate section before selecting a response.

Materials & experimental systems

n/a	Involved in the study	
☐	☒	Antibodies
☐	☒	Eukaryotic cell lines
☒	☐	Palaeontology
☐	☒	Animals and other organisms
☐	☒	Human research participants
☒	☐	Clinical data

Methods

n/a	Involved in the study	
☐	☒	ChIP-seq
☐	☒	Flow cytometry
☒	☐	MRI-based neuroimaging

Antibodies

Antibodies used

PE Rat Anti-Mouse GR-1 (RB6-8C5) BD 553128
RRID:AB_394644
PE-Cy7 Rat Anti-Mouse CD11b (M1/70) BD 561098
RRID:AB_2033994
PE-Cy7 Rat Anti-Mouse CD117 (2B8) BD 558163 RRID:AB_647250
PE Rat Anti Mouse Sca-1 (D7) BD 553108
RRID:AB_394629

APC Mouse Lineage Cocktail BD 558074
RRID:AB_1645213

BV421 Rat Anti-Mouse CD34 (RAM34) BD 562608
RRID:AB_11154576

PerCP-e710 Rat Anti-Mouse CD16/32 (93) eBiosciences 46-0161-80 RRID:AB_2016632

e450 Rat Anti-Mouse CD34 (RAM34) eBiosciences 48-0341-80 RRID:AB_2043838
Polyclonal Rabbit Anti-H3K4me1 Abcam ab8895 RRID:AB_306847

Polyclonal Rabbit Anti-H3K4me3 Abcam ab8580 RRID:AB_306649

Polyclonal Rabbit Anti-H3K27ac Abcam ab4729 RRID:AB_2118291

Validation	Primary antibodies were validated by manufacturer.

Eukaryotic cell lines

Policy information about cell lines

Cell line source(s)	293T17 (ATCC), HoxB8-ER immortalized mouse progenitors (A generous gift from David Sykes), CEBPA/CSF3R immortalized mouse bone marrow progenitors (this study).
Authentication	Cell lines were not authenticated in this study as the vast majority of lines used in this study were murine (limited authentication services available) or derived in this study.
Mycoplasma contamination	All cell lines used in this study tested negative for mycoplasma contamination.
Commonly misidentified lines (See ICLAC register)	None

Animals and other organisms

Policy information about studies involving animals; ARRIVE guidelines recommended for reporting animal research

Laboratory animals	Wild type C57BL/6J mice (JAX # 000664), Wild Type Balb/cJ mice (JAX# 000651), Rosa26 ERT2-Cre mice (JAX #008463) and MX-1 Cre mice (003556), CEBPAFlox/Flox mice (JAX # 006447) were obtained from The Jackson Laboratories. Female mice were used for experimentation between 6 and 20 weeks of age, and were age and weight matched in all experiments.
Wild animals	None
Field-collected samples	None
Ethics oversight	The studies contained herein were approved by OHSU IACUC

Note that full information on the approval of the study protocol must also be provided in the manuscript.

Human research participants

Policy information about studies involving human research participants

Population characteristics	We have 63 individual clinical covariates for 993 patients used in this study. These data are provided in the TARGET data matrix: https:// ocg.cancer.gov/programs/target/data-matrix and cited in the Online Methods section Data Availability.
Recruitment	All subject samples were obtained by member COG institutions after written consent was obtained from the parents/guardians of minors upon enrolling in the trial.
Ethics oversight	The study was overseen by the Institutional Review Board at Fred Hutchinson Cancer Research Center (protocol 1642, IR file no. 5236)

Note that full information on the approval of the study protocol must also be provided in the manuscript.

ChIP-seq

Data deposition

☒ Confirm that both raw and final processed data have been deposited in a public database such as GEO.

☒ Confirm that you have deposited or provided access to graph files (e.g. BED files) for the called peaks.

Data access links<br>*May remain private before publication.*	The following secure token has been created to allow review of record GSE122166 while it remains in private status: ynchuouebrsxpoz
Files in database submission	GSM3457082 Empty-vector-replicate.1<br>GSM3457083 Empty-vector-replicate.2<br>GSM3457084 Empty-H3K4me1-ChIP-replicate.1<br>GSM3457085 Empty-H3K4me1-ChIP-replicate.2<br>GSM3457086 Empty-H3K4me3-ChIP-replicate.1<br>GSM3457087 Empty-H3K4me3-ChIP-replicate.2<br>GSM3457088 Empty-INPUT<br>GSM3457089 CSF3R.T618I-H3K27ac-ChIP-replicate.1

GSM3457090 CSF3R.T618I-H3K27ac-ChIP-replicate.2
GSM3457091 CSF3R.T618I-H3K4me1-ChIP-replicate.1
GSM3457092 CSF3R.T618I-H3K4me1-ChIP-replicate.2
GSM3457093 CSF3R.T618I-H3K4me3-ChIP-replicate.1
GSM3457094 CSF3R.T618I-H3K4me3-ChIP-replicate.2
GSM3457095 CSF3R.T618I-INPUT
GSM3457096 CEBPA.v314vw-H3K27ac-ChIP-replicate.1
GSM3457097 CEBPA.v314vw-H3K27ac-ChIP-replicate.2
GSM3457098 CEBPA.v314vw--H3K4me1-ChIP-replicate.1
GSM3457099 CEBPA.v314vw-H3K4me1-ChIP-replicate.2
GSM3457100 CEBPA.v314vw-H3K4me3-ChIP-replicate.1
GSM3457101 CEBPA.v314vw-H3K4me3-ChIP-replicate.2
GSM3457102 CEBPA.v314vw-INPUT
GSM3457103 CSF3R.T618I+CEBPA.v314vw-H3K27ac-ChIP-replicate.1
GSM3457104 CSF3R.T618I+CEBPA.v314vw-H3K27ac-ChIP-replicate.2
GSM3457105 CSF3R.T618I+CEBPA.v314vw-H3K4me1-ChIP-replicate.1
GSM3457106 CSF3R.T618I+CEBPA.v314vw-H3K4me1-ChIP-replicate.2
GSM3457107 CSF3R.T618I+CEBPA.v314vw-H3K4me3-ChIP-replicate.1
GSM3457108 CSF3R.T618I+CEBPA.v314vw-H3K4me3-ChIP-replicate.2
GSM3457109 CSF3R.T618I+CEBPA.v314vw-INPUT

Genome browser session
(e.g. UCSC)

https://genome.ucsc.edu/cgi-bin/hgTracks?
hgS_doOtherUser=submit&hgS_otherUserName=braunt&hgS_otherUserSessionName=Mousemm10%20HoxB8%
20Chromatin%20States

Methodology

Replicates

All ChIP seq experiments were performed in duplicate. Peaks that were discordant between replicates were discarded

Sequencing depth

75 bp SE sequencing was utilized for all experiments.
Sample ID ChIP Raw reads Alignment efficiency Reads aligned post quality filter (MapQ>30) % Reads surviving alignment/filtering No. of peaks
E1 H3K4me1 40,789,667 98.87% 36,034,794 88 86,665
E1 H3K4me3 23,122,328 97.75% 19,288,141 83 24,574
E1 H3K27ac 22,559,904 98.85% 19,557,264 87 53,213
E1 input 26,202,989 99.50% 19,928,496 76
E2 H3K4me1 45,311,914 97.94% 38,871,346 86 77,076
E2 H3K4me3 24,649,365 98.67% 20,351,411 83 21,957
E2 H3K27ac 22,753,151 99.10% 19,807,300 87 52,404
T/V1 H3K4me1 40,776,454 99.08% 35,814,880 88 104,662
T/V1 H3K4me3 21,234,075 98.54% 17,645,893 83 22,849
T/V1 H3K27ac 24,110,563 99.22% 21,234,517 88 59,747
T/V1 input 49,502,125 99.45% 37,577,725 76
T/V2 H3K4me1 40,632,658 99.14% 35,799,766 88 103,976
T/V2 H3K4me3 22,826,044 98.40% 19,684,656 86 23,612
T/V2 H3K27ac 26,637,730 99.30% 23,323,911 88 62,881
T1 H3K4me1 41,516,693 99.04% 36,099,452 87 79,075
T1 H3K4me3 23,694,704 98.70% 19,516,665 82 20,944
T1 H3K27ac 24,005,869 99.21% 20,897,258 87 53,544
T2 H3K4me1 42,015,447 99.11% 35,303,987 84 66,095
T2 H3K4me3 19,713,772 98.05% 16,501,523 84 22,292
T2 H3K27ac 22,530,642 99.17% 19,653,773 87 51,282
T2 input 23,982,821 99.57% 18,219,744 76
V1 H3K4me1 43,087,545 98.81% 37,512,566 87 98,713
V1 H3K4me3 25,654,067 98.29% 21,441,138 84 21,353
V1 H3K27ac 22,592,515 99.00% 19,553,119 87 49,257
V2 H3K4me1 38,686,465 99.17% 32,597,794 84 87,094
V2 H3K4me3 22,582,654 98.61% 18,674,799 83 19,086
V2 H3K27ac 22,821,405 98.89% 19,512,701 86 45,857
V2 input 47,916,198 99.43% 36,530,722 76

Antibodies

H3K4me1 (ab8895, Abcam), H3K4me3 (ab8580, Abcam) and H3K27ac (ab4729, Abcam)

Peak calling parameters

Peaks were called using MACS2 v2.1.1 using the following parameters:
control file =E1-input-ECP19.30qual-filter.sorted.bam
effective genome size = 1.87e+09
band width = 300
model fold = [5, 50]
qvalue cutoff for narrow/strong regions = 5.00e-02
qvalue cutoff for broad/weak regions = 1.00e-01
Larger dataset will be scaled towards smaller dataset.
Range for calculating regional lambda is: 1000 bps and 10000 bps

Broad region calling is on
Paired-End mode is off

Data quality

Reads were aligned to the mouse reference genome (mm10) using bwa 0.7.1236 with default single end settings. Low mapping alignments were removed with samtools (MAPQ<30). The following peaks of enrichment were found with q<0.05 for each sample.
Sample ID No. of peaks
E1 86,665
E1 24,574
E1 53,213
E1
E2 77,076
E2 21,957
E2 52,404
T/V1 104,662
T/V1 22,849
T/V1 59,747
T/V1
T/V2 103,976
T/V2 23,612
T/V2 62,881
T1 79,075
T1 20,944
T1 53,544
T2 66,095
T2 22,292
T2 51,282
T2
V1 98,713
V1 21,353
V1 49,257
V2 87,094
V2 19,086
V2 45,857
V2

Software

Raw read qualities were inspected using FastQC. Reads were aligned to the mouse reference genome (mm10) using bwa 0.7.12 with default single end settings. Low mapping alignments were removed with samtools (MAPQ<30). Next, MACS2 2.1.1 was used to predict significant peaks of ChIP-seq enrichment relative to the appropriate input controls and generate fold enrichment tracks. We used the ChromHMM software to characterize and annotate the genomes of each treatment group according to 6 chromatin states To construct a catalogue of all putative active enhancers, we calculated a union of all regions annotated as active enhancers across treatment groups and merged enhancers less than 500bp apart. Using BEDOPS, we identified the closest gene to each enhancer in the union peak catalogue and we used BEDTools.To assess differences in levels of H3K4me1 across treatments, we used MACS2 bdgdiff subcommand. Motif enrichment and peak overlap was determined using HOMER.

Flow Cytometry

Plots

Confirm that:

☒ The axis labels state the marker and fluorochrome used (e.g. CD4-FITC).

☒ The axis scales are clearly visible. Include numbers along axes only for bottom left plot of group (a 'group' is an analysis of identical markers).

☒ All plots are contour plots with outliers or pseudocolor plots.

☒ A numerical value for number of cells or percentage (with statistics) is provided.

Methodology

Sample preparation	Mouse bone marrow was subjected to ACK lysis prior to flow staining. 1X10^6 cells were stained in 100 uL buffer
Instrument	FACS sorting was performed using a FACSAria III (BD) using FACSdiva software.
Software	Data was collected using FACSdiva (BD). Flow cytometry data was analyzed using FCSExpress
Cell population abundance	Routine purity checks were performed on GFP/RFP flow sorts which revealed >95% purity.
Gating strategy	For sorting of GFP/RFP positive cell populations, live cells cells were gated on FSC/SCC. Two sequential doublet discriminators were then employed using FSC-W and SCC-W with cells above or below the diagonal being excluded. Gates for GFP/RFP were

determined using single color control cell lines with the threshold for each FP being set using a cell line expressing the other FP. Analytical assessment of mouse bone marrow stem/progenitor cell populations is detailed in Figure S3. Gates were determined based on population morphology, single color controls, unstained controls and FMOs

☒ Tick this box to confirm that a figure exemplifying the gating strategy is provided in the Supplementary Information.
